# Supplementary material for: Understanding Cancer Survivors’ Needs and Experiences Returning to Work Post-Treatment: A Longitudinal Qualitative Study
Source: Curr Oncol. 2022 Apr 23;29(5):3013–25. doi: 10.3390/curroncol29050245 (PMC9139703; doi:10.3390/curroncol29050245)
Supplement: Supplementary file 1 [file curroncol-29-00245-s001.zip › curroncol-1651123-supplementary.pdf]

Draft interview guide for Interview 1. Guides for Interviews 2 and 3 to be drafted based on Interview 1 findings and Feuerstein's cancer and work model.

Looking back

1. Let's start by discussing a little about your cancer and treatment.
   What type of cancer [did/do] you have? What type of treatment did you receive?
   Did you experience any complications or side effects due to your treatment, either short term or more long-lasting? If so, what were they?

2. Tell me about your job before your diagnosis. [Probes: hours, demands, activities]

3. Describe your experience taking a leave of absence after your diagnosis.
   When did you leave?
   What was your employer's reaction? Your co-workers'?
   Have you been in contact with anyone from work since you left? If so, how have these contacts occurred/progressed?

4. Do you have insurance to help you cover some of the financial costs related to your cancer and your leaving work? If so, what type of coverage does it entail? [Probes: long or short term disability, EI sick benefits, CPP disability]
   How has the insurance company reacted to your diagnosis / absence from work?
   Has it been supportive/non-supportive? How so?

Looking forward

5. Tell me about your plans to return to work.
   What are your reasons for returning? [Probes: Return to normalcy, quality of life, finances, identity/work is a part of who you are]
   What does your family think of this decision? [Supportive/non-supportive?]
   Do you ever question this decision? [Why? Why not?]

6. What are some of things you are looking forward to when returning to work?
   What are some things you are a little apprehensive or concerned about? [Probes: energy, concentration, work demands, supervisor reaction, managing symptoms]

7. What do you expect will happen when you return to work? [Probes: your feelings, your family's feelings/reactions, supervisor/co-worker reactions, job stress, support, work changes, work accommodations]

8. Has your cancer care team talked about return to work with you?
   If so, what guidance or resources have you been given?

9. What types of supports or resources do you need – or that might help you – in your transition back to work? [Probes: assistance with paperwork or with HR department, education on what to expect, flexibility to change hours/tasks, support person]

10. Is there anything we have not talked about that you believe is important? If so, what?
